# Leukemic Stem Cells and Hematological Malignancies

**DOI:** 10.3390/ijms25126639

**Published:** 2024-06-17

**Authors:** Hee-Seon Choi, Byoung Soo Kim, Sik Yoon, Sae-Ock Oh, Dongjun Lee

**Affiliations:** 1 Department of Convergence Medicine, School of Medicine, Pusan National University, Yangsan 50612, Republic of Korea; hare177@naver.com; 2 School of Biomedical Convergence Engineering, Pusan National University, Yangsan 50612, Republic of Korea; bskim7@pusan.ac.kr; 3 Department of Anatomy, School of Medicine, Pusan National University, Yangsan 50612, Republic of Korea; sikyoon@pusan.ac.kr (S.Y.); hedgehog@pusan.ac.kr (S.-O.O.); 4 Transplantation Research Center, Research Institute for Convergence of Biomedical Science and Technology, Pusan National University Yangsan Hospital, Yangsan 50612, Republic of Korea

**Keywords:** leukemic stem cell, hematological malignancy, leukemia, hematopoietic stem cell

## Abstract

The association between leukemic stem cells (LSCs) and leukemia development has been widely established in the context of genetic alterations, epigenetic pathways, and signaling pathway regulation. Hematopoietic stem cells are at the top of the bone marrow hierarchy and can self-renew and progressively generate blood and immune cells. The microenvironment, niche cells, and complex signaling pathways that regulate them acquire genetic mutations and epigenetic alterations due to aging, a chronic inflammatory environment, stress, and cancer, resulting in hematopoietic stem cell dysregulation and the production of abnormal blood and immune cells, leading to hematological malignancies and blood cancer. Cells that acquire these mutations grow at a faster rate than other cells and induce clone expansion. Excessive growth leads to the development of blood cancers. Standard therapy targets blast cells, which proliferate rapidly; however, LSCs that can induce disease recurrence remain after treatment, leading to recurrence and poor prognosis. To overcome these limitations, researchers have focused on the characteristics and signaling systems of LSCs and therapies that target them to block LSCs. This review aims to provide a comprehensive understanding of the types of hematopoietic malignancies, the characteristics of leukemic stem cells that cause them, the mechanisms by which these cells acquire chemotherapy resistance, and the therapies targeting these mechanisms.

## 1. Introduction

The development of red blood cells, which are responsible for oxygen exchange and iron metabolism in the body, and immune cells, which mount the inflammatory response to pathogens and cancers, is regulated by hematopoietic stem cells (HSCs) maintained in the bone marrow [1]. HSCs are characterized by their ability to self-renew and differentiate into blood and immune cells [2]. HSCs are highly regulated by their niches, such as the osteoblastic niche, vascular niche, and complex signaling pathways [3].

Hematological malignancies, also known as blood cancers, can arise from the abnormal differentiation of HSCs, affecting the development of white blood cells, red blood cells, or platelets [4]. These cancers disrupt normally regulated hematopoiesis, causing an uncontrollable increase in abnormally differentiated cells that outnumber normal HSCs. Genetic and epigenetic mutations; exposure to environmental toxins; and chronic inflammatory responses such as stress, inflammation, and cancer therapy can lead to the development of blood cancer [5,6]. Hematological malignancies are classified into three categories: leukemia, lymphoma, and multiple myeloma. Leukemia includes acute myeloid leukemia, chronic myeloid leukemia, acute lymphocytic leukemia, and chronic lymphocytic leukemia. Acute myeloid leukemia and chronic myeloid leukemia are the most common hematological malignancies in adults [7,8]. Acute lymphocytic leukemia and chronic lymphocytic leukemia are characterized by abnormal growth and the accumulation of immature lymphocytes [9,10]. Lymphoma has two subcategories: Hodgkin lymphoma and non-Hodgkin lymphoma. Hodgkin lymphoma is the most common of the lymphomas. It is derived from B cells that have lost their phenotype, affecting peripheral lymph nodes and the liver, lung, and bone marrow organs [11]. Non-Hodgkin lymphoma includes highly heterogeneous cancer and is mostly derived from B lymphocytes. Some are derived from T lymphocytes and NK lymphocytes. Non-Hodgkin’s lymphomas include diffuse large B-cell lymphoma (DLBCL), follicular lymphoma (FL), mantle cell lymphoma, and Burkitt’s lymphoma [12].

Leukemic stem cells (LSCs) represent a minority population with chemoresistance and the potential to initiate leukemia [13]. The LSCs that cause leukemia are characterized by excessive proliferation and self-renewal, outpacing normal HSCs in replenishing the cell population [14,15,16,17,18,19,20]. In 1994, CD34^+^CD38^−^ cells like HSCs were identified in NOD-SCID mice, demonstrating their ability to induce leukemia [21]. CD34 is expressed on HSCs, LSCs, and bone marrow progenitor cells. CD38 is widely recognized as a marker of myeloid differentiation and is also present on various non-myeloid cell types [22,23,24]. However, relying solely on the described cell surface markers proves challenging due to the heterogeneous nature of leukemic stem cells. Furthermore, additional identifying markers are necessary for monitoring changes in LSC phenotype during and after patient treatment. LSCs are also associated with abnormal signal pathways and dysregulated cytokines. Activation of the Wnt signaling pathway induces the proliferation of LSCs [25]. CXCL12, which plays a role in maintaining homeostasis of HSCs, and its receptor, CXCR4, are excessively expressed on the surface of LSCs [26,27,28]. This maintains LSCs in a quiescent state and confers chemoresistance [29]. Overexpression of CXCR4 can also be induced by HIF-1α (hypoxia-inducible factor 1 subunit alpha), induced by hypoxia in leukemia [30]. Increased production of nitric oxide by LSCs leads to vascular damage [31]. VEGF signaling also contributes to vascular damage, where downstream effectors such as NF-κB, Akt, Erk, HSP90, and Bcl-2 influence the proliferation and survival of leukemia cells [32,33,34]. These signaling pathways and cytokines that are activated or dysregulated in LSCs are associated with immune evasion, proliferation, and chemoresistance of LSCs.

Standard therapy for acute myeloid leukemia (AML) is chemotherapy with cytarabine and anthracycline to treat malfunctioning DNA and RNA production [35]. Various signaling systems are dysregulated in LSCs and are treated with chemotherapeutic agents that target them. To inhibit signaling pathways in LSCs, drugs that target cell surface markers on LSCs and inhibit their function have been approved and are currently used in therapy [36]. However, relapses are frequent because of the resistance and evasion of LSCs. Indeed, in patients with AML who achieve complete remission after drug administration, LSCs that are not eliminated multiply again and cause leukemia to recur [37]. Therefore, to eradicate LSCs that are refractory to standard therapies, a profound understanding of their characteristics and the signaling systems involved in their recurrence and survival is needed. We synthesized the latest research trends regarding LSCs and hematological malignancies to overcome these challenges.

## 2. Hematological Malignancies

**Figure 1:**
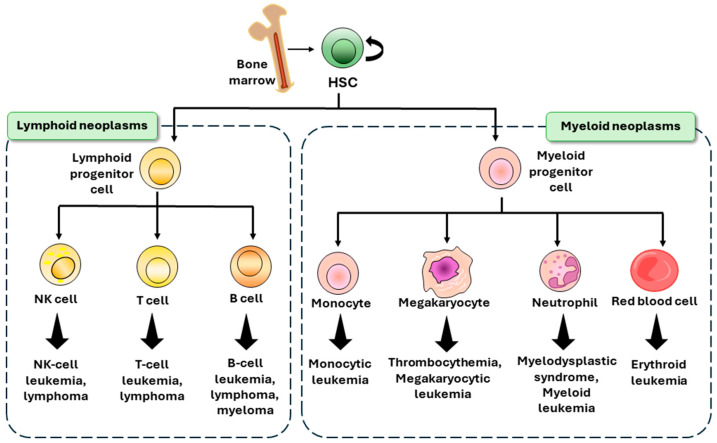
Subtypes of hematological malignancies. Graphical overview of hematological malignancy subtypes according to the HSC hierarchy. HSCs are differentiated into two types of progenitor cells, lymphoid progenitor cells and myeloid progenitor cells [38]. When HSCs are affected by stressors, genetic mutations, viral infections, or cancer therapy which induces abnormal hematopoiesis, they undergo aberrant differentiation, producing abnormal blood cells and hematological malignancies [39]. HSC: hematopoietic stem cell.

Hematological malignancies originate in the bone marrow at the site of hematopoiesis [18]. During viral infection, chronic inflammation, and cancer, the development of aberrant blood cells disrupts the growth and functioning of regular blood cells [18]. Hematological cancer, often known as blood cancer, is distinct from solid cancer because it does not form tumors in organs. Instead, the cancer cells spread throughout the body via the bloodstream. Hematological malignancies can be categorized as leukemia, lymphoma, or multiple myeloma [19] (Figure 1). Leukemia is a type of cancer that affects the blood cells and threatens the regulation of homeostasis [20]. Certain leukemic cells exhibit a rapid growth rate compared to that of other cells [21]. Lymphoma is a condition that arises when lymphocytes undergo aberrant development and transform into lymphoma cells [22]. These cells proliferate rapidly in lymph nodes and other tissues. Typical types of lymphomas include Hodgkin lymphoma, non-Hodgkin lymphoma, AIDS-related lymphoma, and primary central nervous system lymphoma [23,24]. Multiple myeloma is a malignancy that originates from plasma cells [25]. In their normal state, plasma cells generate antibodies. Nevertheless, atypical myeloma disrupts the process of antibody generation and diminishes the immunological response [26].

### 2.1. Leukemia

Leukemia is a hematological disorder characterized by the malfunction and excessive growth of leukocytes [40]. The classification of this condition is based on the level of cell differentiation, with two main categories: acute and chronic [41]. They can be further classified as myeloid or lymphoid [42].

Acute lymphoblastic leukemia (ALL) is a manifestation of the abnormal proliferation of white blood cells originating from lymphoid cells, deviating from their typical differentiation pathways into T or B cells [43]. This type of leukemia is prevalent among younger individuals, making up 76% of cases in children. ALL is characterized by chromosomal abnormalities and genetic alterations linked to the development and proliferation of lymphoid progenitor cells. Representative genetic mutations such as the Philadelphia chromosome-positive ALL in B-cell acute leukemia is a type of leukemia characterized by the presence of the Philadelphia chromosome, which includes the BCR::ABL1 fusion protein formed by the t(9;22)(q34;q11) translocation [44]. The Philadelphia chromosome is found in patients with CML (chronic myeloid leukemia) and ALL. In ALL, the mRNA encoding the P185^BCR::ABL^ protein is expressed [45]. Chromosome translocation of ETV6::RUNX1 also induces the ALL [46]. Among adults, 75% of cases originate from progenitors of B-cell lineage, whereas the remaining cases are composed of malignant T-cell progenitors [47].

Acute myeloid leukemia (AML) is defined as the rapid growth of immature myeloid cells and is the most prevalent type of leukemia, making up 31% of leukemia cases in adults [48,49]. This leads to the proliferation of undifferentiated blast cells in the bone marrow, resulting in the development of aberrant progenitors of bone marrow-lineage stem cells (such as red blood cells, platelets, white blood cells, B cells, and T cells) and subsequent bone marrow dysfunction. The identification of cells unable to reconstitute the HSC pool in immunodeficient mice proved the existence of LSCs [21,50,51]. Through this, it was discovered that AML arises from a pool of LSCs possessing a self-renewal capability [18]. AML is characterized by genetic alterations associated with blood cell formation [48]. These genetic changes lead to the growth of identical undifferentiated bone marrow cells in the blood and bone marrow, causing the generation of aberrant red blood cells and bone marrow failure. A recent study indicated that this may result from a sequence of recurring genetic alterations in HSCs that develop over time [49].

Chronic lymphocytic leukemia (CLL) is a gradually advancing condition in which mature aberrant lymphocytes infiltrate healthy cells in the lymph nodes [52,53,54]. CLL, which accounts for about 30% of leukemia diagnosed in Europeans and North Americans, is more rarely seen among East Asians, Africans, and Latin Americans, exhibiting a racial bias in its occurrence [55]. The lymphocyte count increases in the bloodstream, bone marrow, and lymph nodes, with accumulating CD5+ B cells [52] spreading the disease to the liver and spleen, causing their enlargement [53]. Most cases typically manifest in individuals aged 60–70 years. Most patients have 13q14.3 chromosome mutations, including the deletion of 13q, 11q, or 17p [56]. Recent whole-exome sequencing studies have identified mutated genes in CLL, including *NOTCH1*, *MYD88*, *TP53*, and *ARID1A* [54].

Chronic myeloid leukemia (CML) is a type of leukemia characterized by the excessive growth of myeloid cells in the bone marrow and buildup in the blood [57]. About 30% of cases are diagnosed in adults [8]. This condition is caused by the malfunction of HSCs, which leads to the overproduction of mature granulocytes. Specifically, the *ABL* gene located on chromosome 9 and the *BCR* gene on chromosome 22 undergo translocation, forming BCR::ABL [58]. Unlike in ALL, the transcript encoding the P210^BCR::ABL^ tyrosine kinase is expressed [45]. BCR::ABL regulates the cell cycle, promotes cell division, and hinders DNA repair, resulting in CML development. BCR::ABL exhibits tyrosine kinase activity; therefore, the tyrosine kinase inhibitor imatinib is a therapeutic intervention for CML, primarily affecting individuals aged 25–60 [59].

### 2.2. Lymphoma

Lymphoma is characterized by the aberrant development of lymphocytes, resulting in the formation of lymphoma cells [12,60,61,62]. These cells cluster together in the lymph nodes or other organs. A lymphoma is a neoplasm that arises in the craniofacial region and originates from the lymphocytic system. Malignant lymphomas are categorized as Hodgkin lymphoma and non-Hodgkin lymphoma. Hodgkin lymphoma is a hematopoietic neoplasm, also known as B-cell lymphoma [60]. Hodgkin lymphoma is characterized by Reed–Sternberg cells, which are derived from abnormal B cells and have acquired immunoglobulin variable chain gene mutations. Classical Hodgkin lymphoma has four subtypes: nodular sclerosis classical Hodgkin lymphoma, mixed cellularity, lymphocyte-rich classical Hodgkin lymphoma, and lymphocyte-depleted classical Hodgkin lymphoma [61]. This classification is associated with Epstein–Barr virus infection. Non-Hodgkin lymphoma represents approximately 5% of malignant tumors that develop in the head and neck region [62]. Non-Hodgkin lymphoma arises from diverse differentiation stages of lymphocytes and is a subtype of B-cell, T-cell, and NK-cell lymphomas. The risk factors for non-Hodgkin lymphoma have been widely studied and are thought to include genetic mutations, immune disorders, viral and bacterial infections, and obesity [12]. While research on the contribution of LSCs to the onset and formation of lymphoma is not well-established, it is known that, in an abnormal environment where the expression of key transcription factors such as GATA2 (GATA-binding factor 2) is not regulated in HSCs, reprogramming of Hodgkin lymphoma, a type of lymphoma, can be induced by Hodgkin and Reed–Sternberg (HRS) cells [60].

The treatment for lymphoma consists of hematopoietic stem cell transplantation; radiation therapy; chemotherapies; and targeted drug therapy, such as immune checkpoint inhibitors [63]. The standard treatment for lymphoma combines radiation therapy and chemotherapy. For Hodgkin lymphoma, regimens such as ABVD (doxorubicin, bleomycin, vinblastine, and dacarbazine) and BEACOPP (bleomycin, etoposide, doxorubicin, cyclophosphamide, vincristine, procarbazine, and prednisone) are used [64]. The addition of methotrexate to CHOP (cyclophosphamide, doxorubicin, vincristine, and prednisone) for non-Hodgkin lymphoma is associated with a high failure rate, and treatments such as rituximab are ineffective for relapsed and refractory patients [65]. Therefore, chemotherapeutic drugs targeting relapsed and refractory patients, such as Zanubrutinib, are being researched, and alternative treatment modalities besides chemotherapy are also being explored [66]. CAR (Chimeric antigen receptor) T cells and bispecific antibodies are being applied to lymphoma treatments. Axicabtagene ciloleucel, used in CAR T-cell therapy, has been effective in refractory B-cell lymphoma patients [67].

### 2.3. Multiple Myeloma

Plasma cells generate antibodies that target and combat antigens [68]. Multiple myeloma is a hematological malignancy characterized by the aberrant differentiation and proliferation of plasma cells [68]. Myeloma cells proliferate, leading to the formation of tumors, infiltration of the bone marrow, and subsequent depletion of red and white blood cells. Genetic anomalies in oncogenes such as *CMYC*, *NRAS*, and *KRAS* may affect the growth of plasma cells; however, the specific cause remains unclear [69,70,71]. Multiple myeloma stem cells, like cancer stem cells or leukemic stem cells, are functionally and phenotypically heterogeneous and induce resistance to chemotherapy and radiation therapy [72]. CAR T-cell or bispecific-antibody therapy is also being applied to treat multiple myeloma. A recent study shows that GPRC5D is a G protein-coupled receptor and a target for multiple myeloma treatment. Talquetamab targets GPRC5D and has shown efficacy in refractory multiple myeloma patients [73].

## 3. LSCs and Phenotype

Leukemia is defined as an aggressive hematologic malignancy, and LSCs can contribute to relapse [18]. AML is characterized by the excessive production of dysfunctional leukemic cells replenished by LSCs [13]. Leukemia cells, particularly AML cells, represent an LSC subtype that originates from hematopoietic stem and progenitor cells (HSPCs). LSCs share functional and molecular properties with HSPCs [74]. Leukemia cells arise from a pool of LSCs with the potential for rapid proliferation. Thus, LSCs have the potential for self-renewal and imperfect differentiation, leading to leukemia recurrence. In the 1990s, cells expressing CD34 and lacking CD38, like HSCs, were discovered in NOD-SCID mice, demonstrating their capacity to induce leukemia [18,21]. CD34 is present in HSCs, LSC, and bone marrow progenitor cells. CD38 is a common marker for myeloid differentiation and is also expressed in several types of non-myeloid cells [22,23,24]. Compared with normal HSCs, LSCs are enriched in the CD34^+^CD38^−^ population [75]. However, relying solely on these surface markers poses challenges due to the heterogeneous nature of leukemic stem cells. For instance, LSCs derived from NPM-mutated AML often exhibit low CD34 expression, with certain samples containing CD34^−^ LSCs, while others display a combination of CD34^+^ and CD34^−^ cells [76,77]. Stem cell activity is also detected within the CD34^+^CD38^+^ cells [74,78]. Therefore, the phenotype of LSCs varies considerably. Moreover, additional markers are required to monitor changes in LSC phenotype during and after patient treatment. Some AML LSC surface markers, including CD34, CD38, CD71, and HLA-DR, overlapped with the normal HSC surface markers [79]. This makes it difficult to distinguish between normal HSCs and LSCs, hindering the categorization of LSC-targeted therapies.

To facilitate the precise distinction between HSCs and LSCs, additional markers, including CD25, CD32, CD44, CD96, CD123, G protein-coupled receptor56 (GPR56), IL-1 receptor accessory protein (IL1RAP), T-cell immunoglobulin and mucin domain-containing protein 3 (TIM3), C-type lectin-like molecule-1 (CLL-1), and CD47, have been identified through various studies [19,20,80,81,82,83,84,85]. Apart from cell surface markers, factors such as ALDH (aldehyde dehydrogenase), histone deacetylase (HDAC), and HIF-1α can further enhance the distinction of LSCs [22]. AML relapse is caused by a subclone in which the LSCs are not eliminated or are not detected at the time of diagnosis. LSCs that relapse and survive undergo phenotypic changes and acquire additional mutations.

## 4. Effect of the Hematopoietic Microenvironment on LSCs

Crosstalk between multiple bone marrow cells regulates HSCs [86]. These interaction mechanisms are essential for retaining HSCs and interrupting malignant cell processes. Genetic and epigenetic changes in HSCs and bone marrow niche remodeling can cause hematological malignancy [87,88]. In addition, alterations in bone marrow niches contribute to leukemogenesis and progression. LSCs reside in the bone marrow immune microenvironment [89]. This microenvironment provides LSCs with protection against apoptosis, provides resistance to leukemia therapy, and circumvents immune responses [90]. For example, bone marrow stromal cells can stabilize the β-catenin, which is associated with resistance to tyrosine kinase inhibitors used to treat CML [91]. Wnt signaling maintains HSC homeostasis [92]. The activation of the Wnt-ß catenin pathway is related to the self-renewal capacity of LSCs. In CML, BCR::ABL1 stimulates β-catenin expression [93]. Interference with the BCR::ABL–PI3K–AKT pathway can suppress the transcription of β-catenin and consequently reduce the occurrence of CML [93]. β-catenin mutation in osteoblasts activates the Notch signaling pathway [94]. After the progression of leukemia, LSCs gradually change depending on Wnt signaling [95]. Lower JunB expression has been detected in many types of leukemia [96]. This suggests that LSCs are attenuated by Notch and TGF-β [96,97]. Altered microenvironments affect hematological malignancies in non-mutated hematopoietic cells. The deletion of the ubiquitin E3 ligase Mib1 regulates Wnt-3A-mediated activation [98], suppresses Notch signaling, and consequently causes myeloproliferative neoplasms [99].

The FMS-like tyrosine kinase 3–internal tandem duplications (FLT3-ITD) mutation is common in AML [100]. Remodeling of the hematopoietic niche under hematopoietic malignancy caused by this mutation decreases levels of normal HSCs via tumor necrosis factor and decreases levels of mesenchymal stem cells (MSCs) and endothelial cells [101]. In one study, Pim1 kinase activity in FLT-ITD mutants improved CXCR4 and chemokine receptor signaling [102,103]. Rock1 expression is dysregulated in FLT-ITD+ leukemic cells that migrate to CXCL12, which is highly expressed in the bone marrow niche [104]. A lack of IκBα dysregulates myelopoiesis [105], which could lead to an increase in colony-forming unit granulocyte/erythroid/monocyte/macrophage hyper-granulopoiesis and proliferation.

The CXCL12–CXCR4 axis is also important for maintaining the LSC pool [29,106,107]. CXCL12-deficient MSCs upregulate the LSC cell cycle and are sensitive to tyrosine kinase inhibitors [106]. CXCL12 receptor and CXCR4 overexpression in CML cells enhances their proliferation capacity and resistance to chemotherapy [29]. In CML, a reduction in the homing and retention of long-term HSCs (LTHSCs) in the bone marrow leads to enhanced LTHSC differentiation and immature progenitor expansion. Additionally, a decrease in CXCL12, which regulates LTHSC quiescence in the bone marrow, is related to increased G-CSF production by leukemia cells and alters chemokine and cytokine secretion [107].

LSC senescence and niche remodeling can cause resistance to therapy [108]. Overexpression of the *RAB27B* gene, which regulates exosome expression, is associated with poor prognosis in AML patients [109]. Alterations in the bone marrow microenvironment can protect LSCs and allow them to acquire chemotherapy resistance.

## 5. Role of LSCs in Leukemogenesis and Relapse

The excessive generation of leukemic blast cells is induced by LSCs [110,111]. Rapid cell proliferation and replenishment cause relapse and chemoresistance. Moreover, t(8;21) chromosome-translocated AML forms a RUNX1-ETO fusion protein that disrupts normal RUNX1 activity [110]. Recent studies using t(8;21) AML patient-derived xenograft models revealed that abnormal vascular endothelial growth factor (VEGF) and IL-5 signaling promoted LSC proliferation [111].

LSCs can remodel the bone marrow microenvironment via normal hematopoiesis [32,33,112,113,114]. AML induces bone marrow vascular remodeling via nitric oxide (NO) production. Elevated NO levels can lead to the development of porous vasculatures [32]. VEGF can be used to remodel the vascular niche. Compared with normal bone marrow, VEGF-A and VEGF-C protein levels are higher in the bone marrow of patients with AML [112]. The activation of factors downstream of VEGF signaling, such as NF-κB, Akt, Erk, HSP90, and Bcl-2, induces AML cell proliferation and survival [33,113]. LSCs that interact with vascular niches are associated with AML recurrence [114].

**Figure 2:**
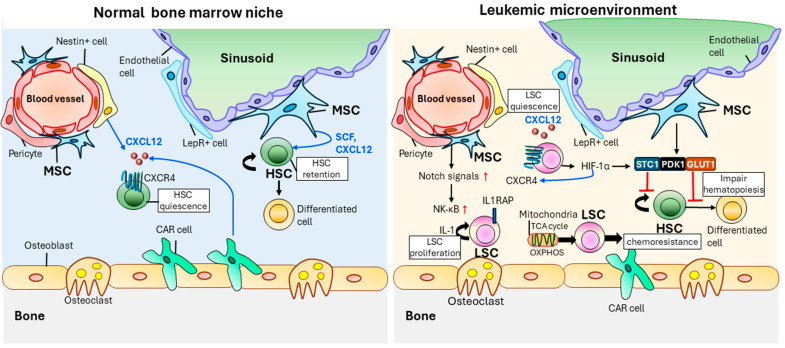
Alteration of MSC function via LSCs in the bone marrow niche. MSC, CXCR12-abundant T cells (CAR cells), and leptin receptor cells in the bone marrow niche play a role in the maintenance of HSCs [38]. However, under the influence of leukemia, LSCs can alter MSC function. MSCs, Nestin^+^ cells, leptin receptor cells, and CAR cells are dysregulated in leukemia [117,118]. AML cell-derived HIF-1α induces the secreted factors, including STC1, PDK1, and GLUT1, and can impair hematopoiesis and protect LSCs [119]. HIF-1α induces increased CXCR4 expression on the surface of leukemic stem cells and can maintain LSC quiescence and protect LSCs through the CXCR4-CXCL12 axis [120,121,122]. Upregulated Notch signals in MSCs suppress apoptosis [123]. Also, the activation of Notch signals elevates NF-κB activation [124]. IL-1 expression induces inflammatory environments [125]. IL1RAP is highly expressed in LSCs to elevate proliferation [126]. MSC supports LSC survival and chemoresistance through upregulated OXPHOS and TCA cycle in mitochondria [127,128]. MSC: mesenchymal stem cell. HSC: hematopoietic stem cell. LSC: leukemic stem cell. HIF-1α: hypoxia-inducible factor 1 subunit alpha. STC1: stanniocalcin-1. PDK1: phosphoinositide-dependent protein kinase 1. GLUT1: glucose transporter 1. IL-1: interleukin 1. IL1RAP: IL-1 receptor accessory protein. VEGF-A: vascular endothelial growth factor A. VEGF-C: vascular endothelial growth factor C. NF-κB: nuclear factor kappa light-chain enhancer of activated B cells. LepR^+^ cell: leptin receptor^+^ cell. OXPHOS: oxidative phosphorylation.

LSCs can affect MSCs and alter the interactions between MSCs and bone marrow niches [38,115] (Figure 2). MSC maturation occurs more frequently in myeloproliferative neoplasms than in healthy MSCs [115]. Bone marrow MSCs, CXCR12-abundant reticular cells, and leptin receptor cells contribute to maintaining HSC homeostasis [26,38]. However, in the leukemic microenvironment, CXCR4, a CXCL12 receptor that is critical for maintaining HSCs, is abnormally overactivated by AML cells [116].

MSCs acquire chemotherapy resistance by transferring mitochondria and increasing oxidative phosphorylation (OXPHOS) and the TCA cycle, which can help them manage increased AML-derived NADPH oxidase 2 (NOX2) and ROS (reactive oxygen species (ROS) levels [127,128]. In the leukemic niche, cytokines, chemokines, and inflammatory factors are aberrantly expressed in LSCs, influencing leukemic cells’ survival and growth [108]. LSCs and MSCs interact via cytokine networks [119,126,129,130]. The overexpression of *STC1*, *PDK1*, and *GLUT1* in MSCs impairs hematopoiesis and supports LSCs [119]. MSCs elevate Notch signaling and repress apoptosis to protect AML cells [129]. Notch activation also contributes to NF-kB activation [130]. IL-1 expression in LSCs constitutes another pro-inflammatory environment [125,131]. IL1RAP is abnormally expressed in LSCs. Dysregulated IL1RAP suppresses AML cell proliferation and increases apoptosis [126].

Both LSCs and HSCs reside in the hypoxic bone marrow niche [132]. Hypoxic conditions are favorable for maintaining HSC function to defend the antioxidant and HSC quiescent state with low ROS levels [133]. LSCs compete with HSCs in the bone marrow microenvironment. A hypoxic state promotes the production of HIF-1α [134]. This upregulates CXCR4 expression on the LSC surface. The CXCR4–CXCL12 axis makes LSCs quiescent in the bone marrow niche. This provides LSCs with protective shelters and enhances resistance to chemotherapy. Hypoxia also retains quiescence in LSCs but does not suppress growth [135]. The mTOR and HIF-1α pathways are correlated with chemoresistance by hypoxia [135]. mTORC1 and its downstream 4EBP1 and S6R phosphorylation are suppressed by HIF-1α in hypoxic T-cell acute lymphoblastic leukemia (T-ALL). T-ALL cells pretreated with the mTORC1 protein kinase inhibitor rapamycin have low sensitivity to chemotherapy [135].

## 6. Clinical Applications of LSCs

Despite achieving complete remission through chemotherapy, patients exhibit high recurrence rates and poor prognosis. Therefore, novel therapeutic strategies are needed. Exhausted HSCs can be prevented from maintaining a quiescent state [136]. Similar to HSCs, leukemic cells, including LSCs, acquire a quiescent state [21]. Indeed, upon isolating stem cells from patients with AML and implanting them into NOD/SCID mice, researchers noted that a significant proportion demonstrated the ability to undergo renewed proliferation [137]. LSCs are highly heterogeneous cells that acquire quiescent, dividing, and senescent phases. Because chemotherapy targets rapidly proliferating cells, quiescent LSCs are resistant to therapy. Quiescent LSCs also upregulate Bcl-2 protein expression and decrease metabolism, leading to drug resistance [138]. Quiescent HSCs depend on glycolysis [138]. Quiescent LSCs produce energy via OXPHOS. High OXPHOS activity in LSCs is associated with cytarabine resistance. OXPHOS-derived products such as NADH+ and FADH_2_ are produced by amino acids or fatty acids that rely on mitochondria. Thus, interference with mitochondrial homeostasis could attenuate LSC function (e.g., venetoclax; Bcl-2 inhibitor) [139]. The combination administration of a Bcl-2 inhibitor and a peroxisome proliferator-activated receptor alpha (PPARα) agonist (chiglitazar) showed an elevated effect on LSC apoptosis [140].

LSC surface markers are potential targets for leukemia treatment [141]. Targeting CD33 using gemtuzumab ozogamicin (GO) decreased relapse and improved the patient survival rate [142]. However, the efficacy of CD33 detection using GO remains unclear because excessive CD33 expression reduces the sensitivity of GO. CD123 is a promising target because of its predominant expression in AML [143]. Anti-CD123 CAR T-cell treatment after pretreatment with 5′-azacitidine exhibits apoptotic activity toward AML cells and induces TNF-α production. Also, this usage does not affect the healthy hematopoietic system [143].

TIM3 is upregulated on the LSCs surface but not in normal HSCs; TIM-3 and galectin 9 (Gal-9), the ligand of TIM3, and the autocrine loop hyperactivate the canonical Wnt and β-catenin signals in LSCs [84]. The constitutive activity of Wnt signaling induces LSCs to renew themselves and multiply [84]. TIM3 is a promising candidate for therapeutic intervention in AML and myelodysplastic syndrome. Sabatolimab targets the T-cell immunoglobulin domain and TIM-3 in LSCs [144]. Sabatolimab is suitable for the treatment of AML, myelodysplastic syndrome, and CML. This drug has been tested in phase 3 clinical trials.

C-type lectin-like molecule-1 (CLL-1) has been discovered in patients with recurrent AML [145]. CLL-1 expression is upregulated compared to healthy HSCs. Thus, CLL-1 can be used as a diagnostic marker and treatment target. Engineering anti-CLL-1 antibodies such as conjugating antibodies and toxic drugs (e.g., pyrrolobenzodiazepine and isoquinolidinobenzodiazepine) or developing chimeric antigen receptors that target CLL-1 may improve clinical efficacy [146,147].

Hypomethylating agents are commonly administered to elderly or refractory AML patients [148,149,150]. In response to hypomethylating agents, LSCs upregulate CD70 expression, resulting in increased CD70/CD27 signaling [151]. CD70 is a tumor necrosis factor receptor ligand that is not expressed in normal hematopoietic cells [152]. Treatment with cusatuzumab, which targets CD70, blocks CD70/CD signaling and has been shown to reduce the number of LSCs in vitro and in vivo [151].

## 7. Conclusions

Recent studies have revealed that HSCs interact with various cells in the bone marrow during proliferation, differentiation, homing, and self-renewal [153,154]. These finely regulated networks are altered by genetic and epigenetic mutations, aging, and environmental factors and affect the development of hematological malignancies. During the initial phase of development, LSCs are like normal HSCs. However, in the subsequent phase, LSCs become home to progenitors and alter bone marrow microenvironment signals.

LSCs are refractory to standard chemotherapy. In 1970, a 7-day course of cytarabine followed immediately by 3 days of anthracycline was the standard of care for AML [155]. Beyond the “7 + 3” administration, novel drugs have been identified as researchers elucidate the signaling pathways and characteristics of LSCs. In CML, asciminib, which targets BCR::ABL1, is used in patients with refractory and tyrosine kinase inhibitor failure [156]. Avelumab and nivolumab target immune checkpoints with high expression on the LSC surface [157]. Ivosidenib and enasidenib, mutant IDH1 and IDH2 inhibitors, promote leukemic cell differentiation [158,159]. The combination of a BCL-2 inhibitor and venetoclax represses the metabolic dependence of OXPHOS [160].

Medications aimed at cell surface markers exhibiting high expression specifically on LSCs but not HSCs tend to predispose patients to relapse or refractory disease and are therefore increasingly being used in combination with chemotherapy drugs and by engineering CAR T cells and antibodies to increase their effectiveness [161,162]. Thus, LSCs become resistant to traditional treatments for leukemia relapse without achieving complete remission. The characterization of LSCs, including the signaling pathways involved in relapse and the altered bone marrow microenvironment in leukemia, may lead to novel therapeutic strategies.

## Data Availability

Not applicable.

## References

[1] Dzierzak E., Bigas A. (2018). Blood development: Hematopoietic stem cell dependence and independence. Cell Stem Cell.

[2] Wilkinson A.C., Igarashi K.J., Nakauchi H. (2020). Haematopoietic stem cell self-renewal in vivo and ex vivo. Nat. Rev. Genet..

[3] Adams G.B., Scadden D.T. (2006). The hematopoietic stem cell in its place. Nat. Immunol..

[4] Gray T.F., Temel J.S., El-Jawahri A. (2021). Illness and prognostic understanding in patients with hematologic malignancies. Blood Rev..

[5] Hu D., Shilatifard A. (2016). Epigenetics of hematopoiesis and hematological malignancies. Genes Dev..

[6] Wade J.C. (2006). Viral infections in patients with hematological malignancies. ASH Educ. Program Book.

[7] Shimony S., Stahl M., Stone R.M. (2023). Acute myeloid leukemia: 2023 update on diagnosis, risk-stratification, and management. Am. J. Hematol..

[8] Miranda-Filho A., Piñeros M., Ferlay J., Soerjomataram I., Monnereau A., Bray F. (2018). Epidemiological patterns of leukaemia in 184 countries: A population-based study. Lancet Haematol..

[9] Hoelzer D., Gökbuget N. (2003). Acute lymphocytic leukemia in adults. Hematology.

[10] Rozman C., Montserrat E. (1995). Chronic lymphocytic leukemia. N. Engl. J. Med..

[11] Küppers R., Engert A., Hansmann M.-L. (2012). Hodgkin lymphoma. J. Clin. Investig..

[12] Armitage J.O., Gascoyne R.D., Lunning M.A., Cavalli F. (2017). Non-hodgkin lymphoma. Lancet.

[13] Stelmach P., Trumpp A. (2023). Leukemic stem cells and therapy resistance in acute myeloid leukemia. Haematologica.

[14] Palani H.K., Ganesan S., Balasundaram N., Venkatraman A., Korula A., Abraham A., George B., Mathews V. (2024). Ablation of Wnt signaling in bone marrow stromal cells overcomes microenvironment-mediated drug resistance in acute myeloid leukemia. Sci. Rep..

[15] Bütow M., Testaquadra F.J., Baumeister J., Maié T., Chatain N., Jaquet T., Tillmann S., Crysandt M., Costa I.G., Brümmendorf T.H. (2023). Targeting cytokine-induced leukemic stem cell persistence in chronic myeloid leukemia by IKK2-inhibition. Haematologica.

[16] Klement L., Drube J. (2023). The interplay of FLT3 and CXCR4 in acute myeloid leukemia: An ongoing debate. Front. Oncol..

[17] Tremblay C.S., Saw J., Boyle J.A., Haigh K., Litalien V., McCalmont H., Evans K., Lock R.B., Jane S.M., Haigh J.J. (2023). STAT5 activation promotes progression and chemotherapy resistance in early T-cell precursor acute lymphoblastic leukemia. Blood J. Am. Soc. Hematol..

[18] Hope K.J., Jin L., Dick J.E. (2004). Acute myeloid leukemia originates from a hierarchy of leukemic stem cell classes that differ in self-renewal capacity. Nat. Immunol..

[19] Saito Y., Kitamura H., Hijikata A., Tomizawa-Murasawa M., Tanaka S., Takagi S., Uchida N., Suzuki N., Sone A., Najima Y. (2010). Identification of therapeutic targets for quiescent, chemotherapy-resistant human leukemia stem cells. Sci. Transl. Med..

[20] Jin L., Hope K.J., Zhai Q., Smadja-Joffe F., Dick J.E. (2006). Targeting of CD44 eradicates human acute myeloid leukemic stem cells. Nat. Med..

[21] Lapidot T., Sirard C., Vormoor J., Murdoch B., Hoang T., Caceres-Cortes J., Minden M., Paterson B., Caligiuri M.A., Dick J.E. (1994). A cell initiating human acute myeloid leukaemia after transplantation into SCID mice. Nature.

[22] Buss E.C., Ho A.D. (2011). Leukemia stem cells. Int. J. Cancer.

[23] Yang J., Ii M., Kamei N., Alev C., Kwon S.-M., Kawamoto A., Akimaru H., Masuda H., Sawa Y., Asahara T. (2011). CD34^+^ cells represent highly functional endothelial progenitor cells in murine bone marrow. PLoS ONE.

[24] Lund F.E., Cockayne D.A., Randall T.D., Solvason N., Schuber F., Howard M.C. (1998). CD38: A new paradigm in lymphocyte activation and signal transduction. Immunol. Rev..

[25] Sakoda T., Kikushige Y., Miyamoto T., Irifune H., Harada T., Hatakeyama K., Kunisaki Y., Kato K., Akashi K. (2023). TIM-3 signaling hijacks the canonical Wnt/β-catenin pathway to maintain cancer stemness in acute myeloid leukemia. Blood Adv..

[26] Mendelson A., Frenette P.S. (2014). Hematopoietic stem cell niche maintenance during homeostasis and regeneration. Nat. Med..

[27] Singh P., Mohammad K.S., Pelus L.M. (2020). CXCR4 expression in the bone marrow microenvironment is required for hematopoietic stem and progenitor cell maintenance and early hematopoietic regeneration after myeloablation. Stem Cells.

[28] Ramakrishnan R., Peña-Martínez P., Agarwal P., Rodriguez-Zabala M., Chapellier M., Högberg C., Eriksson M., Yudovich D., Shah M., Ehinger M. (2020). CXCR4 signaling has a CXCL12-independent essential role in murine MLL-AF9-driven acute myeloid leukemia. Cell Rep..

[29] Agarwal P., Isringhausen S., Li H., Paterson A.J., He J., Gomariz Á., Nagasawa T., Nombela-Arrieta C., Bhatia R. (2019). Mesenchymal niche-specific expression of Cxcl12 controls quiescence of treatment-resistant leukemia stem cells. Cell Stem Cell.

[30] Skinner A.M., O’Neill S.L., Grompe M., Kurre P. (2008). CXCR4 induction in hematopoietic progenitor cells from Fanca^−/−^,-c^−/−^, and-d2^−/−^ mice. Exp. Hematol..

[31] Testa U., Labbaye C., Castelli G., Pelosi E. (2016). Oxidative stress and hypoxia in normal and leukemic stem cells. Exp. Hematol..

[32] Chand R., Chandra H., Chandra S., Verma S.K. (2016). Role of microvessel density and vascular endothelial growth factor in angiogenesis of hematological malignancies. Bone Marrow Res..

[33] Dias S., Choy M., Alitalo K., Rafii S. (2002). Vascular endothelial growth factor (VEGF)–C signaling through FLT-4 (VEGFR-3) mediates leukemic cell proliferation, survival, and resistance to chemotherapy. Blood J. Am. Soc. Hematol..

[34] Giles F.J. (2001). The vascular endothelial growth factor (VEGF) signaling pathway: A therapeutic target in patients with hematologic malignancies. Oncologist.

[35] Tallman M.S., Gilliland D.G., Rowe J.M. (2005). Drug therapy for acute myeloid leukemia. Blood.

[36] Vetrie D., Helgason G.V., Copland M. (2020). The leukaemia stem cell: Similarities, differences and clinical prospects in CML and AML. Nat. Rev. Cancer.

[37] Medeiros B.C., Chan S.M., Daver N.G., Jonas B.A., Pollyea D.A. (2019). Optimizing survival outcomes with post-remission therapy in acute myeloid leukemia. Am. J. Hematol..

[38] Morrison S.J., Scadden D.T. (2014). The bone marrow niche for haematopoietic stem cells. Nature.

[39] Riether C., Schürch C., Ochsenbein A. (2015). Regulation of hematopoietic and leukemic stem cells by the immune system. Cell Death Differ..

[40] Gilliland D.G., Jordan C.T., Felix C.A. (2004). The molecular basis of leukemia. ASH Educ. Program Book.

[41] Munir A.H., Khan M.I. (2019). Pattern of basic hematological parameters in acute and chronic leukemias. J. Med. Sci..

[42] Arber D.A., Campo E., Jaffe E.S. (2023). Advances in the classification of myeloid and lymphoid neoplasms. Virchows Arch..

[43] Malard F., Mohty M. (2020). Acute lymphoblastic leukaemia. Lancet.

[44] Maino E., Sancetta R., Viero P., Imbergamo S., Scattolin A.M., Vespignani M., Bassan R. (2014). Current and future management of Ph/BCR-ABL positive ALL. Expert Rev. Anticancer Ther..

[45] Clark S.S., McLaughlin J., Timmons M., Pendergast A.M., Ben-Neriah Y., Dow L.W., Crist W., Rovera G., Smith S.D., Witte O.N. (1988). Expression of a distinctive BCR-ABL oncogene in Ph1-positive acute lymphocytic leukemia (ALL). Science.

[46] Mullighan C.G., Collins-Underwood J.R., Phillips L.A., Loudin M.G., Liu W., Zhang J., Ma J., Coustan-Smith E., Harvey R.C., Willman C.L. (2009). Rearrangement of CRLF2 in B-progenitor–and Down syndrome–associated acute lymphoblastic leukemia. Nat. Genet..

[47] Terwilliger T., Abdul-Hay M. (2017). Acute lymphoblastic leukemia: A comprehensive review and 2017 update. Blood Cancer J..

[48] Lagunas-Rangel F.A., Chávez-Valencia V., Gómez-Guijosa M.Á., Cortes-Penagos C. (2017). Acute myeloid leukemia—Genetic alterations and their clinical prognosis. Int. J. Hematol.-Oncol. Stem Cell Res..

[49] Hackl H., Astanina K., Wieser R. (2017). Molecular and genetic alterations associated with therapy resistance and relapse of acute myeloid leukemia. J. Hematol. Oncol..

[50] Larochelle A., Vormoor J., Hanenberg H., Wang J.C., Bhatia M., Lapidot T., Moritz T., Murdoch B., Xiao X.L., Kato I. (1996). Identification of primitive human hematopoietic cells capable of repopulating NOD/SCID mouse bone marrow: Implications for gene therapy. Nat. Med..

[51] Lapidot T., Pflumio F., Doedens M., Murdoch B., Williams D.E., Dick J.E. (1992). Cytokine stimulation of multilineage hematopoiesis from immature human cells engrafted in SCID mice. Science.

[52] Hallek M., Cheson B.D., Catovsky D., Caligaris-Cappio F., Dighiero G., Döhner H., Hillmen P., Keating M., Montserrat E., Chiorazzi N. (2018). iwCLL guidelines for diagnosis, indications for treatment, response assessment, and supportive management of CLL. Blood J. Am. Soc. Hematol..

[53] Stevenson F.K., Forconi F., Kipps T.J. (2021). Exploring the pathways to chronic lymphocytic leukemia. Blood.

[54] Puente X.S., Pinyol M., Quesada V., Conde L., Ordóñez G.R., Villamor N., Escaramis G., Jares P., Beà S., González-Díaz M. (2011). Whole-genome sequencing identifies recurrent mutations in chronic lymphocytic leukaemia. Nature.

[55] Yang S., Varghese A.M., Sood N., Chiattone C., Akinola N.O., Huang X., Gale R.P. (2021). Ethnic and geographic diversity of chronic lymphocytic leukaemia. Leukemia.

[56] Puiggros A., Blanco G., Espinet B. (2014). Genetic abnormalities in chronic lymphocytic leukemia: Where we are and where we go. BioMed Res. Int..

[57] Cortes J., Pavlovsky C., Saußele S. (2021). Chronic myeloid leukaemia. Lancet.

[58] Pane F., Intrieri M., Quintarelli C., Izzo B., Muccioli G.C., Salvatore F. (2002). BCR/ABL genes and leukemic phenotype: From molecular mechanisms to clinical correlations. Oncogene.

[59] Lee H., Basso I.N., Kim D.D.H. (2021). Target spectrum of the BCR-ABL tyrosine kinase inhibitors in chronic myeloid leukemia. Int. J. Hematol..

[60] Küppers R. (2009). The biology of Hodgkin’s lymphoma. Nat. Rev. Cancer.

[61] Townsend W., Linch D. (2012). Hodgkin’s lymphoma in adults. Lancet.

[62] de Leval L., Jaffe E.S. (2020). Lymphoma classification. Cancer J..

[63] Ullah F., Dima D., Omar N., Ogbue O., Ahmed S. (2023). Advances in the treatment of Hodgkin lymphoma: Current and future approaches. Front. Oncol..

[64] Lewis W.D., Lilly S., Jones K.L. (2020). Lymphoma: Diagnosis and treatment. Am. Fam. Physician.

[65] Crombie J., LaCasce A. (2021). The treatment of Burkitt lymphoma in adults. Blood J. Am. Soc. Hematol..

[66] Tam C.S., Opat S., Simpson D., Cull G., Munoz J., Phillips T.J., Kim W.S., Rule S., Atwal S.K., Wei R. (2021). Zanubrutinib for the treatment of relapsed or refractory mantle cell lymphoma. Blood Adv..

[67] Neelapu S.S., Locke F.L., Bartlett N.L., Lekakis L.J., Miklos D.B., Jacobson C.A., Braunschweig I., Oluwole O.O., Siddiqi T., Lin Y. (2017). Axicabtagene ciloleucel CAR T-cell therapy in refractory large B-cell lymphoma. N. Engl. J. Med..

[68] Bhutani M., Foureau D.M., Atrash S., Voorhees P.M., Usmani S.Z. (2020). Extramedullary multiple myeloma. Leukemia.

[69] Cao Y., Shan H., Liu M., Liu J., Zhang Z., Xu X., Liu Y., Xu H., Lei H., Yu M. (2021). Directly targeting c-Myc contributes to the anti-multiple myeloma effect of anlotinib. Cell Death Dis..

[70] Shirazi F., Jones R.J., Singh R.K., Zou J., Kuiatse I., Berkova Z., Wang H., Lee H.C., Hong S., Dick L. (2020). Activating KRAS, NRAS, and BRAF mutants enhance proteasome capacity and reduce endoplasmic reticulum stress in multiple myeloma. Proc. Natl. Acad. Sci. USA.

[71] Perroud C., Thurian D., Andres M., Künzi A., Wiedemann G., Zeerleder S., Bacher U., Pabst T., Banz Y., Porret N. (2023). Effect of MAPK activation via mutations in NRAS, KRAS and BRAF on clinical outcome in newly diagnosed multiple myeloma. Hematol. Oncol..

[72] Huff C.A., Matsui W. (2008). Multiple myeloma cancer stem cells. J. Clin. Oncol. Off. J. Am. Soc. Clin. Oncol..

[73] Rodriguez-Otero P., van de Donk N.W., Pillarisetti K., Cornax I., Vishwamitra D., Gray K., Hilder B., Tolbert J., Renaud T., Masterson T. (2024). GPRC5D as a novel target for the treatment of multiple myeloma: A narrative review. Blood Cancer J..

[74] Eppert K., Takenaka K., Lechman E.R., Waldron L., Nilsson B., Van Galen P., Metzeler K.H., Poeppl A., Ling V., Beyene J. (2011). Stem cell gene expression programs influence clinical outcome in human leukemia. Nat. Med..

[75] Zeijlemaker W., Grob T., Meijer R., Hanekamp D., Kelder A., Carbaat-Ham J.C., Oussoren-Brockhoff Y.J., Snel A.N., Veldhuizen D., Scholten W.J. (2019). CD34^+^ CD38^−^ leukemic stem cell frequency to predict outcome in acute myeloid leukemia. Leukemia.

[76] Taussig D.C., Vargaftig J., Miraki-Moud F., Griessinger E., Sharrock K., Luke T., Lillington D., Oakervee H., Cavenagh J., Agrawal S.G. (2010). Leukemia-initiating cells from some acute myeloid leukemia patients with mutated nucleophosmin reside in the CD34^−^ fraction. Blood J. Am. Soc. Hematol..

[77] Moshaver B., Kelder A., Westra G., van Rhenen A., Ossenkoppele G.J., Zweegman S., Schuurhuis G.J. (2007). Identification of a Small Subpopulation of Candidate Leukemia Initiating Cells within the Side Population (SP) of Patients with Acute Myeloid Leukemia. Blood.

[78] Taussig D.C., Miraki-Moud F., Anjos-Afonso F., Pearce D.J., Allen K., Ridler C., Lillington D., Oakervee H., Cavenagh J., Agrawal S.G. (2008). Anti-CD38 antibody–mediated clearance of human repopulating cells masks the heterogeneity of leukemia-initiating cells. Blood J. Am. Soc. Hematol..

[79] Blair A., Hogge D., Sutherland H. (1998). Most acute myeloid leukemia progenitor cells with long-term proliferative ability in vitro and in vivo have the phenotype CD34^+^/CD71^−^/HLA-DR^−^. Blood J. Am. Soc. Hematol..

[80] Hosen N., Park C.Y., Tatsumi N., Oji Y., Sugiyama H., Gramatzki M., Krensky A.M., Weissman I.L. (2007). CD96 is a leukemic stem cell-specific marker in human acute myeloid leukemia. Proc. Natl. Acad. Sci. USA.

[81] Vergez F., Nicolau-Travers M.-L., Bertoli S., Rieu J.-B., Tavitian S., Bories P., Luquet I., De Mas V., Largeaud L., Sarry A. (2020). CD34^+^ CD38^−^ CD123^+^ leukemic stem cell frequency predicts outcome in older acute myeloid leukemia patients treated by intensive chemotherapy but not hypomethylating agents. Cancers.

[82] Pabst C., Bergeron A., Lavallée V.-P., Yeh J., Gendron P., Norddahl G.L., Krosl J., Boivin I., Deneault E., Simard J. (2016). GPR56 identifies primary human acute myeloid leukemia cells with high repopulating potential in vivo. Blood J. Am. Soc. Hematol..

[83] Mitchell K., Barreyro L., Todorova T.I., Taylor S.J., Antony-Debré I., Narayanagari S.-R., Carvajal L.A., Leite J., Piperdi Z., Pendurti G. (2018). IL1RAP potentiates multiple oncogenic signaling pathways in AML. J. Exp. Med..

[84] Kikushige Y., Miyamoto T. (2015). Identification of TIM-3 as a leukemic stem cell surface molecule in primary acute myeloid leukemia. Oncology.

[85] Yanagisawa B., Perkins B., Karantanos T., Levis M., Ghiaur G., Smith B.D., Jones R.J. (2020). Expression of putative leukemia stem cell targets in genetically-defined acute myeloid leukemia subtypes. Leuk. Res..

[86] Kollet O., Canaani J., Kalinkovich A., Lapidot T. (2012). Regulatory cross talks of bone cells, hematopoietic stem cells and the nervous system maintain hematopoiesis. Inflamm. Allergy-Drug Targets.

[87] Taylor J., Xiao W., Abdel-Wahab O. (2017). Diagnosis and classification of hematologic malignancies on the basis of genetics. Blood J. Am. Soc. Hematol..

[88] Chung Y.R., Schatoff E., Abdel-Wahab O. (2012). Epigenetic alterations in hematopoietic malignancies. Int. J. Hematol..

[89] Tabe Y., Konopleva M. (2014). Advances in understanding the leukaemia microenvironment. Br. J. Haematol..

[90] Patterson S.D., Copland M. (2023). The bone marrow immune microenvironment in CML: Treatment responses, treatment-free remission, and therapeutic vulnerabilities. Curr. Hematol. Malig. Rep..

[91] Eiring A.M., Khorashad J.S., Anderson D.J., Yu F., Redwine H.M., Mason C.C., Reynolds K.R., Clair P.M., Gantz K.C., Zhang T.Y. (2015). β-Catenin is required for intrinsic but not extrinsic BCR-ABL1 kinase-independent resistance to tyrosine kinase inhibitors in chronic myeloid leukemia. Leukemia.

[92] Gurska L.M., Ames K., Gritsman K. (2019). Signaling pathways in leukemic stem cells. Leuk. Stem Cells Hematol. Malig..

[93] Hu J., Feng M., Liu Z.-L., Liu Y., Huang Z.-L., Li H., Feng W.-L. (2016). Potential role of Wnt/β-catenin signaling in blastic transformation of chronic myeloid leukemia: Cross talk between β-catenin and BCR-ABL. Tumor Biol..

[94] Duchartre Y., Kim Y.-M., Kahn M. (2016). The Wnt signaling pathway in cancer. Crit. Rev. Oncol./Hematol..

[95] Lane S.W., Wang Y.J., Lo Celso C., Ragu C., Bullinger L., Sykes S.M., Ferraro F., Shterental S., Lin C.P., Gilliland D.G. (2011). Differential niche and Wnt requirements during acute myeloid leukemia progression. Blood J. Am. Soc. Hematol..

[96] Santaguida M., Schepers K., King B., Sabnis A.J., Forsberg E.C., Attema J.L., Braun B.S., Passegué E. (2009). JunB protects against myeloid malignancies by limiting hematopoietic stem cell proliferation and differentiation without affecting self-renewal. Cancer Cell.

[97] Krause D.S., Fulzele K., Catic A., Sun C.C., Dombkowski D., Hurley M.P., Lezeau S., Attar E., Wu J.Y., Lin H.Y. (2013). Differential regulation of myeloid leukemias by the bone marrow microenvironment. Nat. Med..

[98] Berndt J.D., Aoyagi A., Yang P., Anastas J.N., Tang L., Moon R.T. (2011). Mindbomb 1, an E3 ubiquitin ligase, forms a complex with RYK to activate Wnt/β-catenin signaling. J. Cell Biol..

[99] Kim Y.-W., Koo B.-K., Jeong H.-W., Yoon M.-J., Song R., Shin J., Jeong D.-C., Kim S.-H., Kong Y.-Y. (2008). Defective Notch activation in microenvironment leads to myeloproliferative disease. Blood J. Am. Soc. Hematol..

[100] Müller J.P., Schmidt-Arras D. (2020). Novel approaches to target mutant FLT3 leukaemia. Cancers.

[101] Mead A.J., Neo W.H., Barkas N., Matsuoka S., Giustacchini A., Facchini R., Thongjuea S., Jamieson L., Booth C.A., Fordham N. (2017). Niche-mediated depletion of the normal hematopoietic stem cell reservoir by Flt3-ITD–induced myeloproliferation. J. Exp. Med..

[102] Green A.S., Maciel T.T., Hospital M.-A., Yin C., Mazed F., Townsend E.C., Pilorge S., Lambert M., Paubelle E., Jacquel A. (2015). Pim kinases modulate resistance to FLT3 tyrosine kinase inhibitors in FLT3-ITD acute myeloid leukemia. Sci. Adv..

[103] Czardybon W., Windak R., Gołas A., Gałęzowski M., Sabiniarz A., Dolata I., Salwińska M., Guzik P., Zawadzka M., Gabor-Worwa E. (2018). A novel, dual pan-PIM/FLT3 inhibitor SEL24 exhibits broad therapeutic potential in acute myeloid leukemia. Oncotarget.

[104] Onish C., Mori-Kimachi S., Hirade T., Abe M., Taketani T., Suzumiya J., Sugimoto T., Yamaguchi S., Kapur R., Fukuda S. (2014). Internal tandem duplication mutations in FLT3 gene augment chemotaxis to Cxcl12 protein by blocking the down-regulation of the Rho-associated kinase via the Cxcl12/Cxcr4 signaling axis. J. Biol. Chem..

[105] Rupec R.A., Jundt F., Rebholz B., Eckelt B., Herzinger T., Flaig M.J., Moosmann S., Plewig G., Dörken B., Förster I. (2005). Stroma-mediated dysregulation of myelopoiesis in mice lacking IκBα. Immunity.

[106] Frietsch J.J., Kastner C., Grunewald T.G., Schweigel H., Nollau P., Ziermann J., Clement J.H., La Rosée P., Hochhaus A., Butt E. (2014). LASP1 is a novel BCR-ABL substrate and a phosphorylation-dependent binding partner of CRKL in chronic myeloid leukemia. Oncotarget.

[107] Zhang B., Ho Y.W., Huang Q., Maeda T., Lin A., Lee S.-u., Hair A., Holyoake T.L., Huettner C., Bhatia R. (2012). Altered microenvironmental regulation of leukemic and normal stem cells in chronic myelogenous leukemia. Cancer Cell.

[108] Peng D., Wang H., Li L., Ma X., Chen Y., Zhou H., Luo Y., Xiao Y., Liu L. (2018). miR-34c-5p promotes eradication of acute myeloid leukemia stem cells by inducing senescence through selective RAB27B targeting to inhibit exosome shedding. Leukemia.

[109] Chen Y., Wen J., Li Q., Peng D., Liao C., Ma X., Wang M., Niu J., Wang D., Li Y. (2024). RAB27B-regulated exosomes mediate LSC maintenance via resistance to senescence and crosstalk with the microenvironment. Leukemia.

[110] Ptasinska A., Assi S.A., Martinez-Soria N., Imperato M.R., Piper J., Cauchy P., Pickin A., James S.R., Hoogenkamp M., Williamson D. (2014). Identification of a dynamic core transcriptional network in t (8; 21) AML that regulates differentiation block and self-renewal. Cell Rep..

[111] Kellaway S.G., Potluri S., Keane P., Blair H.J., Ames L., Worker A., Chin P.S., Ptasinska A., Derevyanko P.K., Adamo A. (2024). Leukemic stem cells activate lineage inappropriate signalling pathways to promote their growth. Nat. Commun..

[112] Hou H.-A., Chou W.-C., Lin L.-I., Tang J.-L., Tseng M.-H., Huang C.-F., Yao M., Chen C.-Y., Tsay W., Tien H.-F. (2008). Expression of angiopoietins and vascular endothelial growth factors and their clinical significance in acute myeloid leukemia. Leuk. Res..

[113] Santos S.C.R., Dias S. (2004). Internal and external autocrine VEGF/KDR loops regulate survival of subsets of acute leukemia through distinct signaling pathways. Blood.

[114] Chien M.-H., Ku C.-C., Johansson G., Chen M.-W., Hsiao M., Su J.-L., Inoue H., Hua K.-T., Wei L.-H., Kuo M.-L. (2009). Vascular endothelial growth factor-C (VEGF-C) promotes angiogenesis by induction of COX-2 in leukemic cells via the VEGF-R3/JNK/AP-1 pathway. Carcinogenesis.

[115] Avanzini M., Bernardo M., Novara F., Mantelli M., Poletto V., Villani L., Lenta E., Ingo D., Achille V., Bonetti E. (2014). Functional and genetic aberrations of in vitro-cultured marrow-derived mesenchymal stromal cells of patients with classical Philadelphia-negative myeloproliferative neoplasms. Leukemia.

[116] Mehrpouri M. (2022). The contributory roles of the CXCL12/CXCR4/CXCR7 axis in normal and malignant hematopoiesis: A possible therapeutic target in hematologic malignancies. Eur. J. Pharmacol..

[117] Tan Z., Kan C., Wong M., Sun M., Liu Y., Yang F., Wang S., Zheng H. (2022). Regulation of malignant myeloid leukemia by mesenchymal stem cells. Front. Cell Dev. Biol..

[118] Tabe Y., Konopleva M. (2017). Leukemia stem cells microenvironment. Stem Cell Microenviron. Beyond.

[119] Waclawiczek A., Hamilton A., Rouault-Pierre K., Abarrategi A., Albornoz M.G., Miraki-Moud F., Bah N., Gribben J., Fitzgibbon J., Taussig D. (2020). Mesenchymal niche remodeling impairs hematopoiesis via stanniocalcin 1 in acute myeloid leukemia. J. Clin. Investig..

[120] Frolova O., Samudio I., Benito J.M., Jacamo R., Kornblau S.M., Markovic A., Schober W., Lu H., Qiu Y.H., Buglio D. (2012). Regulation of HIF-1α signaling and chemoresistance in acute lymphocytic leukemia under hypoxic conditions of the bone marrow microenvironment. Cancer Biol. Ther..

[121] Liou A., Delgado-Martin C., Teachey D.T., Hermiston M.L. (2014). The CXCR4/CXCL12 Axis Mediates Chemotaxis, Survival, and Chemoresistance in T-Cell Acute Lymphoblastic Leukemia.

[122] Pillozzi S., Bernini A., Spiga O., Lelli B., Petroni G., Bracci L., Niccolai N., Arcangeli A. (2019). Peptides and small molecules blocking the CXCR4/CXCL12 axis overcome bone marrow-induced chemoresistance in acute leukemias. Oncol. Rep..

[123] Rosati E., Sabatini R., Rampino G., Tabilio A., Di Ianni M., Fettucciari K., Bartoli A., Coaccioli S., Screpanti I., Marconi P. (2009). Constitutively activated Notch signaling is involved in survival and apoptosis resistance of B-CLL cells. Blood J. Am. Soc. Hematol..

[124] Takam Kamga P., Bazzoni R., Dal Collo G., Cassaro A., Tanasi I., Russignan A., Tecchio C., Krampera M. (2021). The role of notch and wnt signaling in MSC communication in normal and leukemic bone marrow niche. Front. Cell Dev. Biol..

[125] Binder S., Luciano M., Horejs-Hoeck J. (2018). The cytokine network in acute myeloid leukemia (AML): A focus on pro-and anti-inflammatory mediators. Cytokine Growth Factor Rev..

[126] Carey A., Edwards D.K., Eide C.A., Newell L., Traer E., Medeiros B.C., Pollyea D.A., Deininger M.W., Collins R.H., Tyner J.W. (2017). Identification of interleukin-1 by functional screening as a key mediator of cellular expansion and disease progression in acute myeloid leukemia. Cell Rep..

[127] Moschoi R., Imbert V., Nebout M., Chiche J., Mary D., Prebet T., Saland E., Castellano R., Pouyet L., Collette Y. (2016). Protective mitochondrial transfer from bone marrow stromal cells to acute myeloid leukemic cells during chemotherapy. Blood J. Am. Soc. Hematol..

[128] Forte D., García-Fernández M., Sanchez-Aguilera A., Stavropoulou V., Fielding C., Martín-Pérez D., López J.A., Costa A.S., Tronci L., Nikitopoulou E. (2020). Bone marrow mesenchymal stem cells support acute myeloid leukemia bioenergetics and enhance antioxidant defense and escape from chemotherapy. Cell Metab..

[129] Kamga P.T., Bassi G., Cassaro A., Midolo M., Di Trapani M., Gatti A., Carusone R., Resci F., Perbellini O., Gottardi M. (2016). Notch signalling drives bone marrow stromal cell-mediated chemoresistance in acute myeloid leukemia. Oncotarget.

[130] Carter B.Z., Mak P.Y., Chen Y., Mak D.H., Mu H., Jacamo R., Ruvolo V., Arold S.T., Ladbury J.E., Burks J.K. (2016). Anti-apoptotic ARC protein confers chemoresistance by controlling leukemia-microenvironment interactions through a NFκB/IL1β signaling network. Oncotarget.

[131] Hemmati S., Haque T., Gritsman K. (2017). Inflammatory signaling pathways in preleukemic and leukemic stem cells. Front. Oncol..

[132] Morikawa T., Takubo K. (2016). Hypoxia regulates the hematopoietic stem cell niche. Pflügers Arch.-Eur. J. Physiol..

[133] Chen Y., Liang Y., Luo X., Hu Q. (2020). Oxidative resistance of leukemic stem cells and oxidative damage to hematopoietic stem cells under pro-oxidative therapy. Cell Death Dis..

[134] Schepers K., Campbell T.B., Passegué E. (2015). Normal and leukemic stem cell niches: Insights and therapeutic opportunities. Cell Stem Cell.

[135] Fahy L., Calvo J., Chabi S., Renou L., Le Maout C., Poglio S., Leblanc T., Petit A., Baruchel A., Ballerini P. (2021). Hypoxia favors chemoresistance in T-ALL through an HIF1α-mediated mTORC1 inhibition loop. Blood Adv..

[136] O’Reilly E., Zeinabad H.A., Szegezdi E. (2021). Hematopoietic versus leukemic stem cell quiescence: Challenges and therapeutic opportunities. Blood Rev..

[137] Guan Y., Gerhard B., Hogge D.E. (2003). Detection, isolation, and stimulation of quiescent primitive leukemic progenitor cells from patients with acute myeloid leukemia (AML). Blood J. Am. Soc. Hematol..

[138] Lagadinou E.D., Sach A., Callahan K., Rossi R.M., Neering S.J., Minhajuddin M., Ashton J.M., Pei S., Grose V., O’Dwyer K.M. (2013). BCL-2 inhibition targets oxidative phosphorylation and selectively eradicates quiescent human leukemia stem cells. Cell Stem Cell.

[139] Grønningsæter I.S., Reikvam H., Aasebø E., Bartaula-Brevik S., Tvedt T.H., Bruserud Ø., Hatfield K.J. (2020). Targeting cellular metabolism in acute myeloid leukemia and the role of patient heterogeneity. Cells.

[140] Xie C., Zhou H., Qin D., Zheng H., Tang Y., Li W., Zhou J., Liu L., Yu X., Duan H. (2023). Bcl-2 inhibition combined with PPARα activation synergistically targets leukemic stem cell-like cells in acute myeloid leukemia. Cell Death Dis..

[141] Sadovnik I., Herrmann H., Blatt K., Eisenwort G., Mueller N., Stefanzl G., Hoermann G., Herndlhofer S., Bauer K., Peter B. (2016). Evaluation of cell surface markers and targets in leukemic stem cells (LSC) reveals distinct expression profiles, unique drug effects, and specific checkpoint regulation in AML LSC and CML LSC. Blood.

[142] Khan N., Hills R.K., Virgo P., Couzens S., Clark N., Gilkes A., Richardson P., Knapper S., Grimwade D., Russell N.H. (2017). Expression of CD33 is a predictive factor for effect of gemtuzumab ozogamicin at different doses in adult acute myeloid leukaemia. Leukemia.

[143] El Khawanky N., Hughes A., Yu W., Myburgh R., Matschulla T., Taromi S., Aumann K., Clarson J., Vinnakota J.M., Shoumariyeh K. (2021). Demethylating therapy increases anti-CD123 CAR T cell cytotoxicity against acute myeloid leukemia. Nat. Commun..

[144] Xu S., Zhang N., Rinne M.L., Sun H., Stein A.M. (2023). Sabatolimab (MBG453) model-informed drug development for dose selection in patients with myelodysplastic syndrome/acute myeloid leukemia and solid tumors. CPT Pharmacomet. Syst. Pharmacol..

[145] Wang J., Wang W., Chen H., Li W., Huang T., Zhang W., Ling W., Lai P., Wang Y., Geng S. (2021). C-type lectin-like molecule-1 as a biomarker for diagnosis and prognosis in acute myeloid leukemia: A preliminary study. BioMed Res. Int..

[146] Jiang Y.-P., Liu B.Y., Zheng Q., Panuganti S., Chen R., Zhu J., Mishra M., Huang J., Dao-Pick T., Roy S. (2018). CLT030, a leukemic stem cell–targeting CLL1 antibody-drug conjugate for treatment of acute myeloid leukemia. Blood Adv..

[147] Laborda E., Mazagova M., Shao S., Wang X., Quirino H., Woods A.K., Hampton E.N., Rodgers D.T., Kim C.H., Schultz P.G. (2017). Development of a chimeric antigen receptor targeting C-type lectin-like molecule-1 for human acute myeloid leukemia. Int. J. Mol. Sci..

[148] Dombret H., Seymour J.F., Butrym A., Wierzbowska A., Selleslag D., Jang J.H., Kumar R., Cavenagh J., Schuh A.C., Candoni A. (2015). International phase 3 study of azacitidine vs. conventional care regimens in older patients with newly diagnosed AML with> 30% blasts. Blood J. Am. Soc. Hematol..

[149] Craddock C., Quek L., Goardon N., Freeman S., Siddique S., Raghavan M., Aztberger A., Schuh A., Grimwade D., Ivey A. (2013). Azacitidine fails to eradicate leukemic stem/progenitor cell populations in patients with acute myeloid leukemia and myelodysplasia. Leukemia.

[150] Al-Ali H.K., Jaekel N., Niederwieser D. (2014). The role of hypomethylating agents in the treatment of elderly patients with AML. J. Geriatr. Oncol..

[151] Riether C., Pabst T., Höpner S., Bacher U., Hinterbrandner M., Banz Y., Müller R., Manz M.G., Gharib W.H., Francisco D. (2020). Targeting CD70 with cusatuzumab eliminates acute myeloid leukemia stem cells in patients treated with hypomethylating agents. Nat. Med..

[152] Nolte M.A., Van Olffen R.W., Van Gisbergen K.P., Van Lier R.A. (2009). Timing and tuning of CD27–CD70 interactions: The impact of signal strength in setting the balance between adaptive responses and immunopathology. Immunol. Rev..

[153] Wilson A., Trumpp A. (2006). Bone-marrow haematopoietic-stem-cell niches. Nat. Rev. Immunol..

[154] Calvi L.M., Link D.C. (2015). The hematopoietic stem cell niche in homeostasis and disease. Blood J. Am. Soc. Hematol..

[155] Lichtman M.A. (2013). A historical perspective on the development of the cytarabine (7 days) and daunorubicin (3 days) treatment regimen for acute myelogenous leukemia: 2013 the 40th anniversary of 7 + 3. Blood Cells Mol. Dis..

[156] Eide C.A., Zabriskie M.S., Stevens S.L.S., Antelope O., Vellore N.A., Than H., Schultz A.R., Clair P., Bowler A.D., Pomicter A.D. (2019). Combining the allosteric inhibitor asciminib with ponatinib suppresses emergence of and restores efficacy against highly resistant BCR-ABL1 mutants. Cancer Cell.

[157] Riether C., Gschwend T., Huguenin A.-L., Schürch C., Ochsenbein A. (2015). Blocking programmed cell death 1 in combination with adoptive cytotoxic T-cell transfer eradicates chronic myelogenous leukemia stem cells. Leukemia.

[158] Yen K., Travins J., Wang F., David M.D., Artin E., Straley K., Padyana A., Gross S., DeLaBarre B., Tobin E. (2017). AG-221, a first-in-class therapy targeting acute myeloid leukemia harboring oncogenic IDH2 mutations. Cancer Discov..

[159] DiNardo C.D., Stein E.M., de Botton S., Roboz G.J., Altman J.K., Mims A.S., Swords R., Collins R.H., Mannis G.N., Pollyea D.A. (2018). Durable remissions with ivosidenib in IDH1-mutated relapsed or refractory AML. N. Engl. J. Med..

[160] Pollyea D.A., Stevens B.M., Jones C.L., Winters A., Pei S., Minhajuddin M., D’Alessandro A., Culp-Hill R., Riemondy K.A., Gillen A.E. (2018). Venetoclax with azacitidine disrupts energy metabolism and targets leukemia stem cells in patients with acute myeloid leukemia. Nat. Med..

[161] Gupta A., Gill S. (2021). CAR-T cell persistence in the treatment of leukemia and lymphoma. Leuk. Lymphoma.

[162] Sheykhhasan M., Manoochehri H., Dama P. (2022). Use of CAR T-cell for acute lymphoblastic leukemia (ALL) treatment: A review study. Cancer Gene Ther..

